# A Case of Transcatheter Aortic Valve Implantation in a Centenarian Patient

**DOI:** 10.1155/2021/6529390

**Published:** 2021-11-18

**Authors:** Salem Badr, Daniel O. Johnsrud, Charnai D. Sherry, Suhail Q. Allaqaband, Eric S. Weiss, Tanvir K. Bajwa

**Affiliations:** ^1^Aurora Cardiovascular and Thoracic Services, Aurora Sinai/Aurora St. Luke's Medical Centers, 2801 W. Kinnickinnic River Parkway, Ste. 880, Milwaukee, WI 53215, USA; ^2^University of Wisconsin School of Medicine and Public Health, 750 Highland Ave., Madison, WI 53726, USA

## Abstract

Calcified aortic stenosis has become the most common form of acquired valvular heart disease in very old patients. Despite this fact, a majority of these patients were turned down by surgery owing to a risk of mortality > 10% in patients older than 90 years. In recent years, transcatheter aortic valve implantation (TAVI) has emerged as a therapeutic option for severe aortic stenosis. However, there is a paucity of data regarding the outcomes of TAVI in patients older than 100 years. We present the oldest patient who has undergone successful TAVI reported in the current literature.

## 1. Introduction

As the population continues to age, calcified aortic stenosis has become the most common form of acquired valvular heart disease in older adults [1]. Although centenarians are still rare in the world population, the United Nations World Population Prospects 2019 report projects that the number of centenarians will increase from 573,000 in 2020 to more than 19 million in 2100 [2]. Both the prevalence and severity of aortic stenosis increase with extreme age, so it is anticipated that the number of centenarians presenting with severe symptomatic aortic stenosis will increase. Without aortic valve replacement, severe symptomatic aortic stenosis drastically reduces quality of life and increases mortality risks. Surgical aortic valve replacement is associated with 10% perioperative mortality in patients who are more than 90 years old [3]. In recent years, transcatheter aortic valve implantation (TAVI) has emerged as a therapeutic option for severe symptomatic aortic stenosis in patients at high surgical risk [4]. Data regarding the effectiveness of TAVI in centenarians are scarce. Here, we present the case of the oldest patient to successfully undergo TAVI reported in the literature.

## 2. Case Presentation

**Figure 1:**
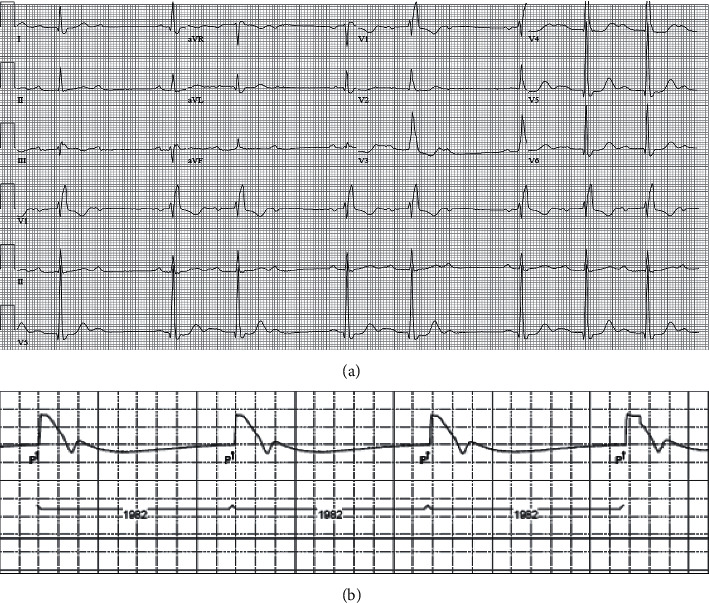
(a) An electrocardiogram performed before transcatheter aortic valve implantation (TAVI) shows sinus rhythm with second-degree AV block (Mobitz type 1), right bundle branch block, and left ventricular hypertrophy with repolarization abnormalities. (b) Post TAVI, a pacemaker check showed sinus rhythm with complete heart block.

**Figure 2:**
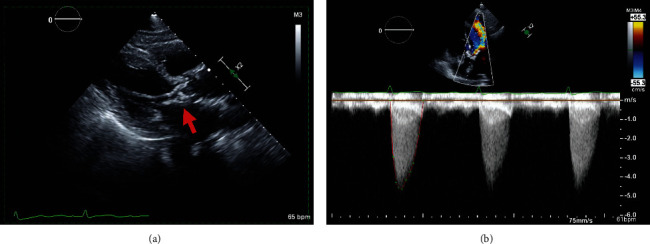
(a) The parasternal long-axis view on transthoracic echocardiography shows bulky calcification of the aortic valve (arrow). (b) The apical five-chamber view on transthoracic continuous-wave Doppler analysis shows a mean gradient of 48 mmHg.

A 103-year-and-9-month-old female patient with a past medical history significant for moderate aortic stenosis since five years prior, hypertension, chronic kidney disease, and hyperlipidemia presented to an outside hospital with 2 weeks of progressive shortness of breath, lower-leg swelling, and hematuria that had started the day before the admission. The patient was independent at baseline and lived by herself. Her blood pressure was 161/72 mmHg, her heart rate was 59 beats per minute, and her respiration rate was 15 breaths per minute. Auscultation of the heart revealed the classic murmur of aortic valve stenosis: a loud, late-peaking ejection murmur with single S2 radiating to the carotid arteries. Lung auscultation revealed diminished air entry and bibasilar rales. She had bilateral lower-extremity edema that was 1+ and nonpitting. The electrocardiogram revealed sinus rhythm with second-degree Mobitz type 1 heart block and the right bundle branch block that had been noted beginning five years prior (Figure 1(a)). A transthoracic echocardiogram revealed severe calcified aortic valve stenosis with an area of 0.78 cm^2^ (normal is 3-4 cm^2^) and a mean transvalvular gradient of 48 mmHg. Mild-to-moderate aortic valve regurgitation (Figure 2) also was seen.

### 2.1. Preoperative Evaluation for TAVI

Subsequently, the patient was transferred to our tertiary care center and evaluated by the heart team. Her preoperative risk assessment for 30-day mortality was intermediate, with a Society of Thoracic Surgeons score of 5.7%. Her frailty score was 4, which means vulnerable (although not reliant on others for daily help, symptoms often limit activities). Multiple tests were performed to assess the feasibility of the TAVI procedure; these included a coronary angiogram that showed diffuse calcification of the coronary arteries without significant stenosis and computed tomographic angiograms of the thorax, abdomen, and pelvis that demonstrated a trileaflet aortic valve with severe valvular calcification (aortic valve calcium score of 1,859 Agatston units) and mild left ventricle outflow calcification. Computed tomography measurements were annulus perimeter, 68 mm; sinus of Valsalva, 27.9 × 29.1 mm; right coronary artery height, 11.8 mm; and left coronary artery height, 12.1 mm. Patent iliofemoral arteries suitable for a transfemoral TAVI approach were noted. Based on high surgical risk scores, age, frailty, and patient comorbidities, the heart team decided to proceed with TAVI using a transfemoral approach.

### 2.2. Performance of TAVI

**Figure 3:**
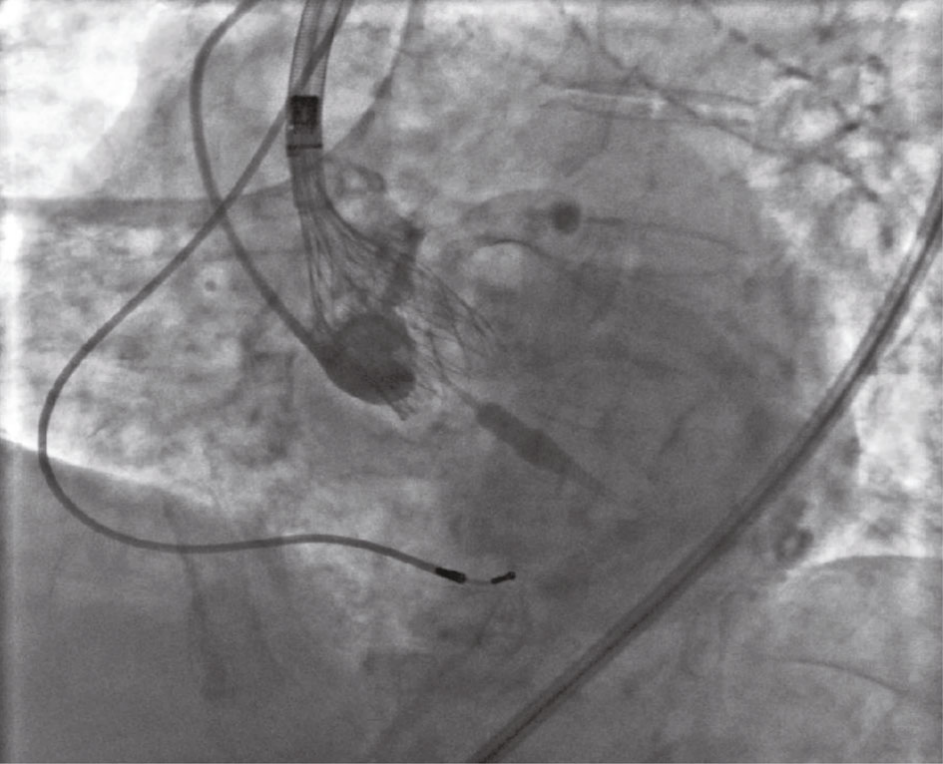
This periprocedural fluoroscopic image shows deployment of a self-expanding TAVI Evolut PRO+ (Medtronic, Minneapolis, MN, USA) device at a depth of 3 mm in the aortic valve during rapid pacing.

Under monitored anesthesia care, the right internal jugular vein and the right femoral artery were each accessed with 6-French sheaths. A temporary pacemaker was placed in the right ventricle through a 6-French sheath in the right internal jugular vein. Two Perclose ProGlide (Abbott Vascular Devices, Santa Clara, CA, USA) suture-mediated devices were employed using the preclosure technique while exchanging the 6-French sheath with a 14-French sheath in the right femoral artery. A 20-mm Z-MED II balloon (Braun International Systems, Melsungen, Germany) was used for predilation of the aortic valve. Subsequently, a self-expanding TAVI 26-mm Evolut PRO+ (Medtronic, Minneapolis, MN, USA) was deployed during rapid pacing at a depth of 3 mm in the aortic valve (Figure 3). This led to a marked improvement of the transvalvular gradient with only mild paravalvular aortic regurgitation. Successful hemostasis was achieved in the right femoral artery using the preclosure technique. There were no intraoperative complications.

### 2.3. Postoperative Course

A 2D echocardiogram performed on the first postoperative day showed that the prosthetic aortic valve was well seated, with a mean transvalvular gradient of 4 mmHg and trace paravalvular regurgitation. No transvalvular regurgitation was seen. A temporary pacemaker check revealed ventricular paced rhythm (99.4%) with underlying sinus rhythm and complete heart block (Figure 1(b)). Subsequently, the patient underwent dual-chamber pacemaker implantation, which she tolerated well without complication. Urology was consulted for intermittent hematuria and recommended observation while she was in the hospital. She was discharged home four days after the TAVI procedure. The patient was seen in the clinic for a 6-month follow-up; she had turned 104 years old and was still doing well. Repeat echocardiography revealed a well-seated and expanded valve with a mean gradient across of 6 mmHg and trace paravalvular regurgitation.

## 3. Discussion

Although centenarians are still rare, the number of centenarians worldwide has grown rapidly over the past 30 years and is projected to increase sixfold by 2050 [2]. Therefore, physicians are expected to encounter more centenarians with severe symptomatic aortic stenosis. TAVI is an attractive treatment modality for centenarians who are not a candidate for surgery due to advanced age and comorbidities [4]. Furthermore, centenarians can paradoxically have greater resilience and fewer comorbidities than younger patients. They can tolerate the TAVI procedure quite well, with low in-hospital and 1-year adverse event rates; however, we acknowledge that this group of patients is underrepresented in the literature and evidence stems only from scarce data that include observational data and one case report [5]. Arsalan et al. reported outcomes for an exceedingly small cohort of centenarians undergoing TAVI. Overall, 24 centenarians who were treated were included in the Society of Thoracic Surgeons/American College of Cardiology Transcatheter Valve Therapy Registry; in this cohort, there was no mortality at 30 days, and an incredibly low 1-year mortality (6.7%) considering the 1-year death probability of centenarians is more than 30% [6]. From the current literature, the oldest patient known to have had TAVI was a 102-year-old woman. In this patient, a 23-mm Edwards Sapien XT valve (Edwards Lifesciences, Irvine, CA, USA) was successfully deployed under general anesthesia using a transfemoral approach. Post TAVI, aortic valve area improved from 0.5 cm^2^ to 1.7 cm^2^, and the patient was discharged about 2 weeks later without any complications. Four years later, a transthoracic echocardiogram revealed the bioprosthetic valve was functioning well, and she was able to perform her daily living activities without support [7].

## 4. Conclusions

We present the oldest patient, to the best of our knowledge, who has undergone successful TAVI. A transfemoral approach was employed under monitored anesthesia care. We believe that in the current TAVI era, age alone should not be a factor in refusing access to this advanced technology. Ultimately, the process of shared decision-making is paramount to ensuring that the course of action is patient-centered and balances the expected risks and benefits of the procedure with the centenarian's preference and values.

## Data Availability

There are no underlying data to report.
